# Contribution of the Testosterone Androgen Receptor–PARD3B Signaling Axis to Tumorigenesis and Malignance of Glioblastoma Multiforme through Stimulating Cell Proliferation and Colony Formation

**DOI:** 10.3390/jcm11164818

**Published:** 2022-08-17

**Authors:** Jr-Di Yang, Jui-Tai Chen, Shing-Hwa Liu, Ruei-Ming Chen

**Affiliations:** 1 Division of Urology, Department of Surgery, National Yang-Ming Chiao Tung University Hospital, Yilan County 260006, Taiwan; 2 Cell Physiology and Molecular Image Research Center, Wan Fang Hospital, Taipei Medical University, Taipei 11696, Taiwan; 3 Department of Anesthesiology, Shuang Ho Hospital, Taipei Medical University, Taipei 23561, Taiwan; 4 Department of Anesthesiology, School of Medicine, College of Medicine, Taipei Medical University, Taipei 11031, Taiwan; 5 Institute of Toxicology, College of Medicine, National Taiwan University, Taipei 10051, Taiwan; 6 Graduate Institute of Medical Sciences, College of Medicine, Taipei Medical University, Taipei 11031, Taiwan; 7 International Ph.D. Program for Cell Therapy and Regeneration Medicine, College of Medicine, Taipei Medical University, Taipei 11031, Taiwan; 8 Anesthesiology and Health Policy Research Center, Taipei Medical University Hospital, Taipei 11031, Taiwan; 9 TMU Research Center of Cancer Translational Medicine, Taipei Medical University, Taipei 11031, Taiwan

**Keywords:** glioblastoma multiforme, testosterone androgen receptor signaling, PARD3B, cell proliferation, colony formation

## Abstract

Background: Glioblastoma multiforme (GBM) is the most common and malignant brain tumor with very poor prognoses. After surgical resection of the primary tumor, rapid proliferation of residual glioblastoma cells is a critical cause explaining tumor malignance and recurrence. In this study, we evaluated de novo roles of the testosterone androgen receptor (AR)–PARD3B signaling axis in the tumorigenesis and malignance of human GBM and the possible related mechanisms. Methods: *AR* and *PARD3B* gene expressions and their correlations were mined from The Cancer Genome Atlas (TCGA) database and analyzed using the UALCAN system. Analyses using a real-time PCR, cell proliferation, and colony formation and a loss-of-function strategy by suppressing AR activity with its specific inhibitor, enzalutamide, were then carried out to determine roles of the testosterone AR–PARD3B signaling axis in tumor malignance. Results: Expressions of AR, PARD3B mRNA, and proteins in human GBM tissues were upregulated compared to normal human brain tissues. In contrast, levels of AR and PARD3B mRNA in most TCGA pan-cancer types were downregulated compared to their respective normal tissues. Interestingly, a highly positive correlation between *AR* and *PARD3B* gene expressions in human GBM was identified. The results of a bioinformatics search further showed that there were five AR-specific DNA-binding elements predicted in the 5′ promoter of the *PARD3B* gene. Regarding the mechanisms, exposure of human glioblastoma cells to testosterone induced *AR* and *PARD3B* gene expressions and successively stimulated cell proliferation and colony formation. Suppressing AR activity concurrently resulted in significant attenuations of testosterone-induced *PARD3B* gene expression, cell proliferation, and colony formation in human glioblastoma cells. Conclusions: This study showed the contribution of the testosterone AR–PARD3B signaling axis to the tumorigenesis and malignance of human GBM through stimulating cell proliferation and colony formation. Therefore, the AR-PARD3B signaling axis could be targeted for potential therapy for human GBM.

## 1. Introduction

Gliomas are the most common primary brain tumors that originate from transformed glial cells [1]. Glioblastoma multiforme (GBM), referred to as a high grade (grade IV) astrocytoma according to classification by the World Health Organization (WHO), is a fast-growing, malignant brain tumor [2]. Currently, standard treatment for GBM patients is surgical resection of the primary tumor followed by concurrent chemo- and radiotherapy (CCRT). However, the prognoses of GBM patients are very poor, with a median survival of as low as 15 months [3]. Rapid proliferation of residual glioblastoma cells after surgical resection of the primary tumor is one of the major reasons explaining tumor malignance and recurrence in GBM. Multiple signaling pathways, such as the Wnt signal, the phosphatidylinositol 3-kinase (PI3K)/Akt/mammalian target of rapamycin (mTOR) axis, and the programmed death (PD)-1 and PD-ligand 1 (PD-L1) axis, are reported to participate in regulating the proliferation of human glioblastoma cells [4,5]. By targeting these signaling pathways, de novo therapeutic strategies can be built up to treat GBM. Dysregulation of cell proliferation is tightly linked to tumorigenesis, malignance, and recurrence of human GBM. As a result, more new mechanisms explaining the proliferation of glioblastoma cells should be explored.

Androgen receptor (AR), a member of the steroid hormone nuclear receptor family, encompasses three major domains for transcriptional regulation and binding to DNA and the ligand [6]. Functionally, following binding with androgens on the ligand-binding domain, the AR can be activated and then triggers sequential intracellular signals for development and maintenance of reproductive, cardiovascular, hemopoietic, musculoskeletal, immune, and neural systems [7]. Furthermore, the AR signaling axis may be involved in the tumorigenesis of prostate, ovary, bladder, lung, liver, and kidney cancers [8,9]. Interestingly, targeting AR signaling has been widely investigated and potentially applied to treat multiple types of cancers, especially prostate cancer [9]. Recently, Łysiak et al. reported the positive correlation of *AR* gene expression with the DNA repair response in the microenvironment of GBM tissues [10]. Expression of the *AR* gene is also positively related to the survival of cancer patients, especially male cases. One possible reason explaining the involvement of AR signaling in the tumorigenesis of glioblastomas is that upregulation of testosterone levels and *AR* gene expression could disrupt the status of hormones and immunity in the microenvironment of glioblastomas and then worsen the outcome of GBM patients [11]. Therefore, the testosterone AR signaling axis may contribute to tumorigenesis and malignance of glioblastomas.

Cell morphologies and shapes are produced through a polarity process [12]. Functionally, cell polarity can determine the division of asymmetric cells, vectorial transport of ions and molecules, and cell proliferation and migration [13]. In tumorigenesis, loss of cell polarity usually leads to invasion and metastasis of cancer cells. Human partitioning defective (Par)-3 family cell polarity regulator (PARD3) and PARD3β (PARD3B) are members of the Par-3 family of proteins that centrally determinate cell polarity and activities [14]. In addition to being a polarity protein, PARD3 can work as a scaffold protein that triggers differential intracellular signals, such as the Hippo-KIBRA/Yap pathway, the PI3K/AKT axis, and the TIAM-mediated Rac1 alliance [15]. Deregulating PARD3-transducing signals consequently induces the proliferation, invasion, and metastasis of cancer cells, ultimately resulting in tumorigenesis and successive malignance and recurrence of tumors. In human epithelial cells, PARD3B also functions as a junction protein for construction of tight junctions [16]. Moreover, PARD3B was reported to contribute to a series of biological events, including cell morphogenesis, proliferation, development, immune response, and cell apoptosis. In colorectal cancer, PARD3B can enhance survival of cancer cells through downregulating the Lkb1-adenosine-monophosphate-activated protein kinase (AMPK) signaling pathway [17]. A case report showed that a novel fusion gene of PARD3B and NUT midline carcinoma family member 1 (NUTM1) was discovered in an aggressive primary central nerve system (CNS) embryonal tumor [18]. Thus, previous studies reported the potential roles of PARD3B in tumorigenesis and malignance. This study was further aimed at evaluating the actions of the testosterone AR–PARD3B signaling axis in the tumorigenesis and malignance of human GBM and the possible related mechanisms.

## 2. Materials and Methods

### 2.1. Data Mining and Analysis

OMICS data of human GBM were mined from The Cancer Genome Atlas (TCGA) database (https://www.cancer.gov/about-nci/organization/ccg/research/structural-genomics/tcga with data downloaded in June 2022 (accessed on 6 June 2022).). All of the data were further analyzed using a tool of the UALCAN database system (http://ualcan.path.uab.edu with data downloaded in June 2022 (accessed on 6 June 2022).) as described previously [19]. In our data mining and analyses, expressions of AR and PARD3B mRNA and proteins were identified. Correlations between expressions of *AR* and *PARD3B* genes were also analyzed using the UALCAN database system. In analyses of proteomic data, Z-value is a measure of how many standard deviations (SDs) below or above the population mean a raw score is. Z-scores are a way to compare results to a “normal” population. Thus, the protein expression was expressed as Z-value in this study. The heatmap was analyzed and obtained from the UALCAN database system as described previously [19]. Briefly, a PERL script was used to analyze normalized TCGA level 3 RNA-seq data for each gene. The mean transcripts per million (TPM) values of each gene in normal and GBM groups were separately obtained. A total of 250 over-expressed genes that possessed significantly different TPM values in GBM tissues from normal brains were selected and listed. Among these genes, only those with median TPM values of 10 or above were retained. Then, a Pearson correlation analysis was carried out using an in-house PERL script that utilizes the “Statistics: Basic” module. Gene pairs showing Pearson correlation coefficients of 0.3 or above were considered positive correlations.

### 2.2. Bioinformatic Approach

The sequence of the AR-specific DNA-binding element contains 5′-GGA/TACANNNTGTTCT-3′ [6]. In this study, the PROMO system was used to predict if the AR-binding element (5′ -GGA/TACANNNTGTTCT-3′) that exists in the 5′ promoter region of the *PARD3B* gene was as previously described [20,21]. Bioinformatics results revealed that there were five predicted AR DNA-binding elements existing in the 5′ promoter of the *PARD3B* gene.

### 2.3. Cell Culture and Drug Treatment

Human U87 MG and GBM8401 glioblastoma cells were respectively purchased from American Type Culture Collection (Manassas, VA, USA) and Bioresource Collection and Research Center (Hsinchu, Taiwan). U87 MG glioblastoma cells were derived from a malignant glioblastoma from a female Caucasian patient [22]. In contrast, GBM8401 glioblastoma cells were isolated from a female Asian GBM patient [23]. Dulbecco’s modified Eagle medium (DMEM, Gibco-BRL, Grand Island, NY, USA) was prepared by supplementation with 10% fetal bovine serum (FBS) inactivated by heating at 50 °C, 2 mM L-glutamine, 100 IU/mL penicillin, 100 mg/mL streptomycin, 1 mM sodium pyruvate, and 1 mM nonessential amino acids. Human glioblastoma cells were seeded in cultured DMEM in an incubator with a humidified atmosphere of 5% CO_2_ at 37 °C [24].

Testosterone was purchased from Sigma-Aldrich (St. Louis, MO, USA). Its purity was >98%. Testosterone was freshly prepared by dissolving it in dimethyl sulfate (DMSO). Our preliminary study showed that testosterone at 1, 10, 100, and 1000 nM time-dependently induced AR mRNA expression in human U87 MG cells. Testosterone at 1000 nM is much higher than clinically relevant concentrations. Thus, human glioblastoma cells were treated with testosterone at 25, 50, 75, and 100 nM for various periods of time. Control cells received DMSO only.

### 2.4. Suppression of AR Activity

To determine the roles of the AR signaling axis in the malignance of human glioblastoma cells, a loss-of-function strategy was carried out via application of enzalutamide, an inhibitor of the AR [25]. Enzalutamide was bought from Sigma-Aldrich, and its purity was >98%. Enzalutamide was dissolved in DMSO. To effectively inhibit AR activity, human U87 MG and GBM8401 cells were pretreated with enzalutamide for 1 h and then exposed to testosterone. Our preliminary results revealed that enzalutamide at 10 μM significantly suppressed the AR activity of human U87 MG glioblastoma cells and did not affect cell viability. Accordingly, enzalutamide at 10 μM was used in this study.

### 2.5. Real-Time Polymerase Chain Reaction (PCR)

A real-time PCR was executed to measure expressions of AR and PARD3B mRNAs in human U87 MG and GBM8401 glioblastoma cells following a previously described methodology [26]. After being exposed to testosterone or enzalutamide, total RNA was isolated from human U87 MG and GBM8401 glioblastoma cells for analyses of AR and PARD3B mRNA. Human AR and PARD3B mRNAs were reverse-transcribed into their complementary (c)DNAs. The upstream and downstream primers of oligonucleotide sequences, designed and synthesized by MDBio (Taipei, Taiwan), were 5′-AGCAACCTTCACAGCCGCAG3′ and 5′-GCTGCTGCTGCCTTCGGATA-3′ for human AR mRNA [27], 5′-CCAGTTGGTGATTCTGAGGAGG-3′ and 5′-TTCGCCTAGAGGTGGTTACAGC-3′ for human PARD3B mRNA (OriGene Technologies, Rockville, MD, USA), and 5′-AGCAACCTTCACAGCCGCAG-3′ and 5′-GCTGCTGCTGCCTTCGGATA-3′ for β-actin mRNA [21]. The real-time PCR was conducted using an iQSYBR Green Supermix kit (Bio-Rad, Hercules, CA, USA), and reactions were carried out using the MyiQ Single-Color Real-Time PCR Detection System (Bio-Rad) as described previously [26]. After enzyme activation at 95 °C for 10 min, the cDNAs of human AR, PARD3B, and β-actin were enlarged with 95 °C for 15 s, 60 °C for 30 s, and 72 °C for 60 s for 40 cycles. The reactions were finally dissociated at 95 °C for 15 s, 60 °C for 60 s, and 95 °C for 15 s and then terminated and maintained at 4 °C.

### 2.6. Cell Proliferation

Proliferation of human U87 MG and GBM8401 glioblastoma cells was determined using a colorimetric method as described previously [28]. A water-soluble tetrazolium (WST)-1 assay kit was purchased from Roche (Mannheim, Germany). Briefly, human glioblastoma cells were seeded in 96-well tissue culture plates at a density of 5 × 10^4^ cells/well overnight. Cells were treated with testosterone or enzalutamide. After drug treatment, WST-1, a red tetrazolium salt, was added to the plates and reacted at 37 °C for 4 h. The yellow products reduced by mitochondrial dehydrogenase were measured using an enzyme-linked immunosorbent assay (ELISA) microplate at an optical density of 450 nm.

### 2.7. Colony Formation

Human U87 MG and GBM8401 glioblastoma cells (5 × 10^5^ cells) were seeded in six-well tissue culture plate at 37 °C overnight. Human glioblastoma cells were treated with testosterone or enzalutamide. After drug treatment, cells were washed with 1× phosphate-buffered saline (PBS) containing NaCl (0.14 M), KH_2_PO_4_ (1.5 mM), Na_2_HPO_4_ (8 mM), and KCl (2.6 mM), and harvested. Human glioblastoma cells were replated in 35 mm tissue culture dishes at a density of 10^3^ cells per dish for 3 days. Colonies were stained with crystal violet, and then the colonies were counted and statistically analyzed as described previously [12].

### 2.8. Statistical Analysis

In normal human brain tissues and GBM tissues, values indicating expressions of various genes at the mRNA and protein levels represent the minimum, lower quartile, median, upper quartile, and maximum. Pearson correlation analyses were conducted to determine the coefficients between *AR* and *PARD3B* gene expressions. In the in vitro studies, each value denotes the mean ± standard error of the mean (SEM) for *n* = 6. Statistical analyses of all data presented in this study were conducted using a two-way analysis of variance (ANOVA) and post-hoc Duncan’s multiple-range test. Differences among various groups were considered significant at *p* < 0.05.

## 3. Results

### 3.1. Upregulation of AR mRNA and Protein Expressions in Human GBM Tissues Compared to Human Normal Brain Tissues

**Figure 1:**
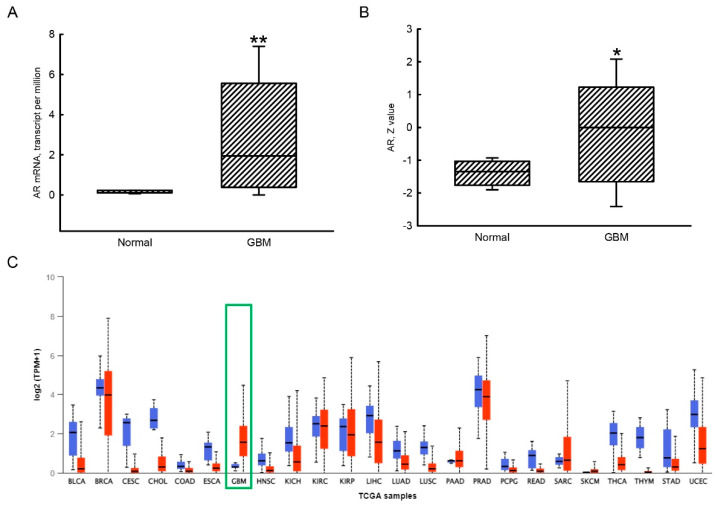
Upregulation of androgen receptor (AR) mRNA and protein expressions in human glioblastoma multiforme (GBM) tissues. Differential expressions of AR mRNA in normal (*n* = 5) and GBM (*n* = 156) tissues were mined from The Cancer Genome Atlas (TCGA) database and analyzed using UALCAN (http://ualcan.path.uab.edu/ (accessed on 6 June 2022).) (**A**). Protein expressions of the AR in normal (*n* = 10) and GBM (*n* = 99) tissues were extracted and analyzed using UALCAN (**B**). Expressions of the *AR* gene across TCGA cancers are shown (**C**). The red boxplot indicates expression levels in primary tumors, while the blue boxplot designates expressions in normal samples. Each value represents the minimum, lower quartile, median, upper quartile, and maximum. The symbols * and ** indicate the values significantly differ from the respective normal group, * *p* < 0.05, ** *p* < 0.01. The GBM is highlighted with a green box.

Compared to normal human brains, levels of AR mRNA in human GBM tissues were massively upregulated by 760% (Figure 1A). Additionally, amounts of the AR protein in human GBM tissues were also elevated in comparison to normal human brain samples (Figure 1B). Compared to GBM tissues, levels of AR mRNA in most TCGA cancer types, including urothelial bladder carcinoma (BLCA), cervical squamous cell carcinoma and endocervical adenocarcinoma (CESC), cholangiocarcinoma (CHOL), squamous cell cancer in the head and neck region (HNSC), kidney chromophobe (KICH), liver hepatocellular carcinoma (LIHC), lung adenocarcinoma (LUAD), lung squamous cell carcinoma (LUSC), rectum adenocarcinoma (READ), thyroid cancer (THCA), tumor mutation burden (TMB) in thymic epithelial tumors (THYM), and uterine corpus endometrial carcinoma (UCEC), were downregulated compared to their respective normal tissues (Figure 1C).

### 3.2. Increased Expression of the PARD3B Gene in GBM Tissues Compared to Human Normal Brain Tissues

**Figure 2:**
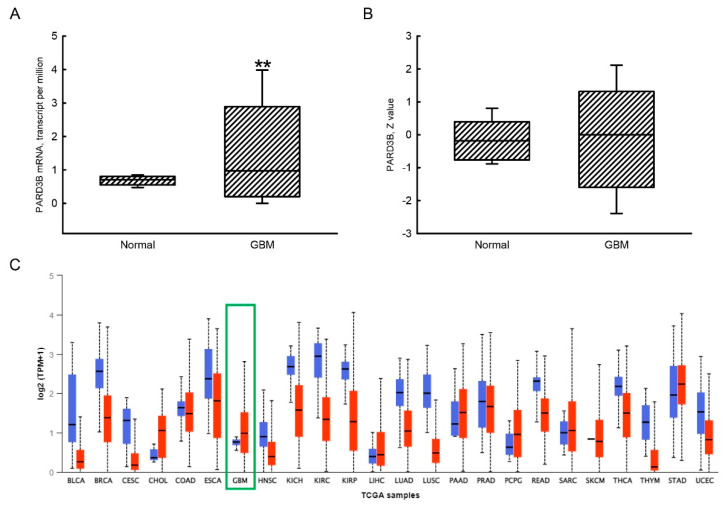
Increased expression of PARD3B mRNA in glioblastoma multiforme (GBM) tissues. Differential expressions of PARD3B mRNA in normal (*n* = 5) and GBM (*n* = 156) tissues were mined from The Cancer Genome Atlas (TCGA) database and analyzed by UALCAN (http://ualcan.path.uab.edu/ (accessed on 6 June 2022).) (**A**). Protein expression of PARD3B in normal (*n* = 18) and GBM (*n* = 125) tissues were mined and analyzed from UALCAN (**B**). Expressions of PARD3B mRNA across TCGA cancers in normal (blue bar) and tumor tissues (red bar) are further shown (**C**). Each value represents the minimum, lower quartile, median, upper quartile, and maximum. The symbol ** indicates that the value significantly differed from the respective normal group, *p* < 0.01.

Results using the UALCAN analytical system revealed low levels of PARD3B mRNA detected in normal human brain tissues (Figure 2A). In contrast, the median expression of PARD3B mRNA in human GBM tissues was enhanced by 40% compared to normal human brain tissues. Basal levels of the PARD3B protein in normal human brain tissues were low but detectable (Figure 2B). In comparison, levels of PARD3B in human GBM tissues were slightly elevated but did not significantly differ from normal human tissues. In addition to human GBM tissues, the levels of PARD3B mRNA in CHOL, PAAD, PCPG, and STAD were upregulated (Figure 2C). Similarly to *AR* gene expression in human GBM tissues, expressions of PARD3B mRNA in most TCGA cancers, including breast invasive carcinoma (BRCA), CESC, HNSC, KICH, KIRC, KIRP, LUAD, LUSC, READ, THCA, THYM, and UCEC, were downregulated (Figure 2C).

### 3.3. A Positive Correlation of the AR Gene Expression with PARD3B Expression in Human GBM Tissues

**Figure 3:**
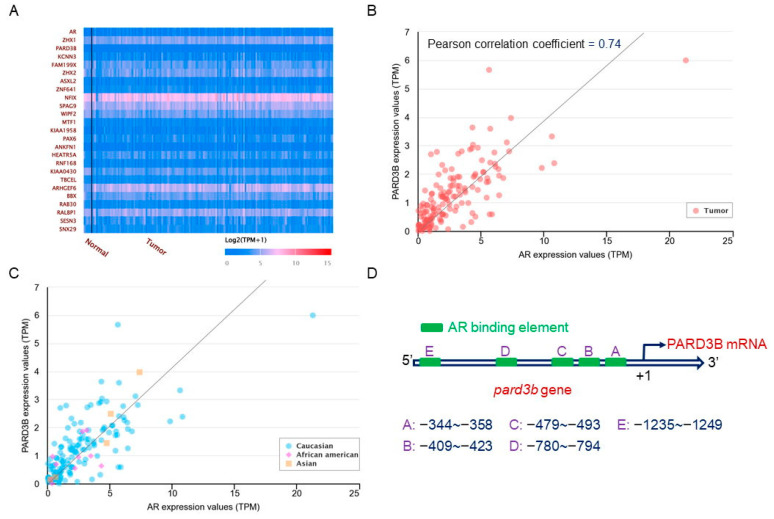
Positive correlation between expressions of androgen receptor (*AR*) and *PARD3B* genes in glioblastoma multiforme (GBM) tissues. A heatmap shows 25 genes that were positively correlated with the *AR* in GBM (**A**). Correlations between expressions of the *AR* and *PARD3B* genes in GBM tissues (*n* = 157) are shown (**B**). Expression correlations of the *AR* and *PARD3B* genes in GBM among Caucasian, African American, and Asian populations were further analyzed (**C**). A bioinformatics search showed that five AR-binding elements were predicted in the 5′ promoter region of the *PARD3B* gene (**D**).

A heatmap in Figure 3A indicates the top 25 gene expressions with highly positive correlations with *AR* gene expression in human GBM tissues. The Pearson correlation coefficients of the *ZHX1*, *PARD3B*, *KCNN3*, *FAM199X*, *ZHX2*, *ASXL2*, *ZNF641*, *NFIX*, *SPAG9*, *WIPF2*, *MTF1*, *KIAA1958*, *PAX6*, *ANKFN1*, *HEATR5A*, *RNF168*, *KIAA0430*, *TBCEL*, *ARHGEF6*, *BBX*, *RAB30*, *RALBP1*, *SESN3*, and *SNX29* genes were 0.75, 0.74, 0.73, 0.72, 0.72, 0.72, 0.71, 0.7, 0.7, 0.7, 0.69, 0.69, 0.69, 0.69, 0.68, 0.68, 0.68, 0.68, 0.67, 0.67, 0.67, 0.67, 0.67, and 0.67, respectively. Moreover, the Pearson correlation coefficient between *AR* and *PARD3B* gene expressions was 0.74 (*n* = 157) (Figure 3B). In Caucasian (*n* = 152), African American (*n* = 10), and Asian (*n* = 5) groups, Pearson correlation coefficients of *AR* and *PARD3B* gene expressions were 0.74, 0.51, and 0.96, respectively (Figure 3C). Results of a bioinformatics search revealed that there were five AR-binding elements in the 5′ promoter area of the *PARD3B* gene, localized to −344–−358, −409–−423, −479–−493, −780–−794, and −1235–−1249 (Figure 3D).

### 3.4. The Testosterone AR Signaling Axis Was Involved in Regulation of PARD3B Gene Expression in Human Glioblastoma Cells

**Figure 4:**
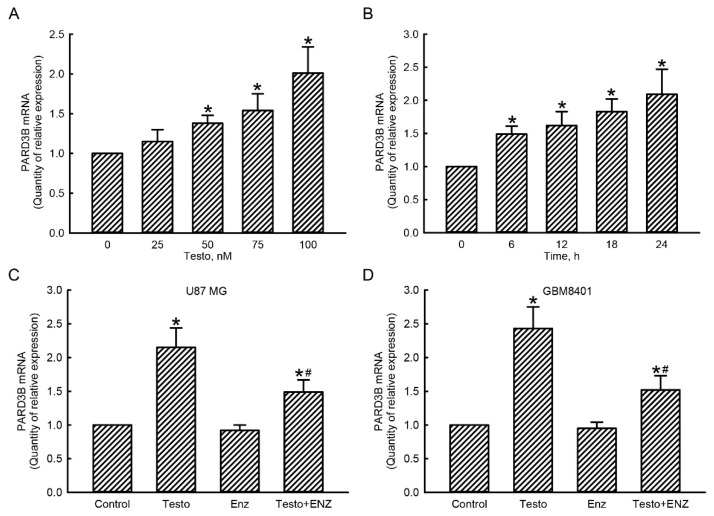
Involvement of the androgen receptor (AR) in regulation of *PARD3B* gene expression in glioblastoma cells. Human U87 MG glioblastoma cells were treated with testosterone (Testo) at 25, 50, 75, and 100 nM for 24 h (**A**) or at 100 nM for 6, 12, 18, and 24 h (**B**). Human U87 MG (**C**) and GBM8401 (**D**) glioblastoma cells were pretreated with 10 μM enzalutamide (Enz) for 1 h and exposed to 100 nM Testo for 24 h. Total RNA was extracted for a real-time PCR analysis of AR mRNA. Each value represents the mean ± standard error of the mean for *n* = 6. The symbols * and # indicate that values significantly (*p* < 0.05) differed from the control and testo-treated groups, respectively.

RNA analyses were carried out to determine the roles of the testosterone AR signaling axis in regulating *PARD3B* gene expression in human glioblastoma cells (Figure 4). Treatment of human U87 MG glioblastoma cells with testosterone at 25 nM for 24 h caused a slight (17%) but non-significant induction in levels of PARD3B mRNA (Figure 4A). However, when the concentration of testosterone reached 50 nM, expression of PARD3B mRNA was significantly increased by 33% in human glioblastoma cells. Exposure of U87 MG glioblastoma cells to 75 and 100 nM testosterone for 24 h led to 50% and 96% augmentations of PARD3B mRNA, respectively (Figure 4A). Treatment of human U87 MG glioblastoma cells with 100 nM testosterone for 6 h induced PARD3B mRNA expression by 50% (Figure 4B). After exposure to 100 nM testosterone for 12, 18, and 24 h, levels of PARD3B mRNA in U87 MG glioblastoma cells were significantly increased by 60%, 85%, and 105%, respectively (Figure 4B).

A loss-of-function strategy was further applied by decreasing AR activity using its specific inhibitor, enzalutamide (Figure 4C,D). Human U87 MG glioblastoma cells were exposed to 100 nM testosterone for 24 h, which caused a significant 115% induction in expression of PARD3B mRNA (Figure 4C). Pretreatment of U87 MG cells with 10 μM enzalutamide did not affect PARD3B mRNA expression. In contrast, pretreatment with enzalutamide led to a 57% inhibition of testosterone-induced PARD3B mRNA in human U87 MG cells (Figure 4C). Exposure of human GBM8401 cells to testosterone increased PARD3B mRNA expression by 145% (Figure 4D). Pretreatment with enzalutamide did not change *PARD3B* gene expression but meaningfully suppressed testosterone-induced PARD3B mRNA expression by 63%.

### 3.5. The Testosterone AR Signaling Axis Contributes to Proliferation of Human Glioblastoma Cells

**Figure 5:**
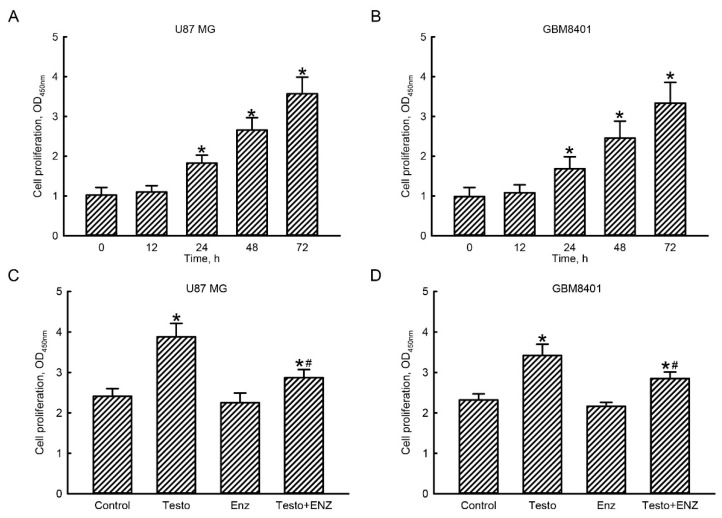
Participation of the testosterone (testo) androgen receptor (AR) signaling axis in proliferation of human glioblastoma cells. Human U87 MG (**A**) and GBM8401 (**B**) glioblastoma cells were treated with 100 nM testo for 12, 24, 48, and 72 h. Human U87 MG (**C**) and GBM8401 (**D**) glioblastoma cells were pretreated with 10 μM enzalutamide (Enz) for 1 h and then exposed to 100 nM testo for 24 h. Proliferation of human glioblastoma cells was assayed using a colorimetric WST-1 method. Each value represents the mean ± standard error of the mean for *n* = 6. The symbols * and # indicate that values significantly (*p* < 0.05) differed from the control and testo-treated groups, respectively.

A colorimetric WST-1 assay was conducted to determine the roles of the testosterone AR signaling axis in the proliferation of human glioblastoma cells (Figure 5). Exposure of human U87 MG glioblastoma cells to 100 nM testosterone for 12 h did not affect cell proliferation (Figure 5A). When the treated time intervals reached to 24, 48, and 72 h, proliferation of human U87 MG glioblastoma cells increased by 75%, 163%, and 250%, respectively. In human GBM8401 glioblastoma cells, exposure to 100 nM testosterone for 12 h did not influence cell proliferation (Figure 5B). However, following exposure for 24, 48, and 72 h, testosterone at 100 nM caused significant 67%, 142%, and 225% enhancements in cell proliferation, respectively (Figure 5B).

Compared to the control group, treatment of human U87 MG glioblastoma cells with 100 nM testosterone for 72 h elevated cell proliferation by 62% (Figure 5C). Pretreatment with 10 μM enzalutamide for 1 h did not affect proliferation of U87 MG glioblastoma cells. In contract, pretreatment with enzalutamide led to a significant 73% suppression of testosterone-induced proliferation of human U87 MG glioblastoma cells (Figure 5C). In human GBM8401 glioblastoma cells, exposure to 100 nM testosterone for 72 h stimulated 41% cell proliferation (Figure 5B). Pretreatment with enzalutamide for 1 h led to a significant 59% attenuation of testosterone-induced proliferation of human GBM8401 glioblastoma cells (Figure 5D).

### 3.6. The Testosterone AR Signaling Axis Contributes to Regulation of Proliferation of Human Glioblastoma Cells

**Figure 6:**
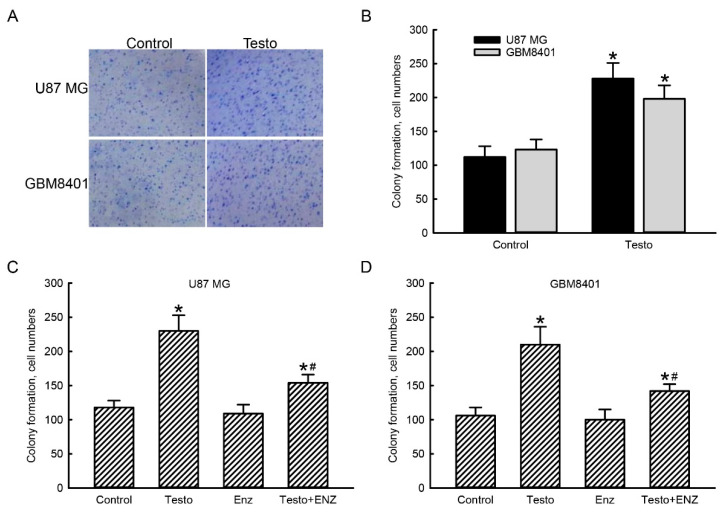
Contribution of the testosterone (testo) androgen receptor (AR) signaling axis to colony formation of human glioblastoma cells. Human U87 MG and GBM8401 glioblastoma cells were treated with 100 nM Testo for 72 h. A colony-formation assay was carried out. Representative cell colony images are shown (**A**). These colonies were quantified and statistically analyzed (**B**). Human U87 MG (**C**) and GBM8401 (**D**) glioblastoma cells were pretreated with 10 μM enzalutamide (Enz) for 1 h and then exposed to 100 nM Testo for 72 h. Colony formation was assayed, quantified, and statistically analyzed. Each value represents the mean ± standard error of the mean for *n* = 6. The symbols * and # indicate that values significantly (*p* < 0.05) differed from the control and testo-treated groups, respectively.

A colony-formation assay was further carried out to determine the roles of the testosterone AR signaling axis in malignance of human GBM (Figure 6). In the control groups, after culturing human U87 MG and GBM8401 glioblastoma cells for 72 h, cell colonies had formed (Figure 6A, left 2 panels). After exposure to 100 nM testosterone for 72 h, colonies of human glioblastoma cells had obviously increased (right two panels). These colonies of human glioblastoma cells were counted and statistically analyzed (Figure 6B). Treatment of human U87 MG and GBM8401 glioblastoma cells with 100 nM testosterone for 72 h led to significant 105% and 69% increases, respectively, in the formation of cell colonies (Figure 6B).

Exposure of human U87 MG glioblastoma cells to 100 nM testosterone for 72 h increased colony formation by 92% (Figure 6C). Pretreatment with enzalutamide did not influence colony formation in human U87 MG cells. In contrast, suppression of AR activity by enzalutamide pretreatment caused a 68% decrease in testosterone-induced colony formation in human U87 MG glioblastoma cells. In comparison, exposure of human GBM8401 glioblastoma cells to 100 nM testosterone for 72 h enhanced colony formation by 100%. Pretreatment with enzalutamide did not affect colony formation but led to a 62% downregulation in testosterone-induced colonized numbers of human GBM8401 glioblastoma cells (Figure 6D).

## 4. Discussion

The testosterone AR-PARD3B axis is involved in malignance of GBM through stimulation of cell proliferation and colony formation. GBM is the most common and aggressive brain tumor [1]. The 5-year relative survival rates of GBM patients among populations of 20–44, 45–54, and 55–64 years of age are 22%, 9%, and 6%, respectively [29]. Causes explaining the malignance and recurrence of GBM are complicated. One of the major reasons is the rapid proliferation, migration, and invasion of residual GBM cells after surgical resection of the tumor site [3]. Our results obtained through data mining revealed upregulated expressions of AR mRNA and protein in human GBM tissues compared to normal human brain tissues. AR can work as an important molecule for developing and maintaining a lot of physiological functions [7]. Furthermore, dysregulation of the AR signaling axis could be involved in tumorigenesis of various types of cancers, including GBM [8,9]. Our present results further demonstrated that in response to stimulation by testosterone, proliferation and colony formation of human U87 MG and GBM5401 glioblastoma cells were significantly enhanced. In contrast, a loss-of-function strategy entailing suppressing AR activity produced significant declines in testosterone-induced cell proliferation and colony formation. These results indicated that the testosterone AR axis participates in proliferation and colony formation of human glioblastoma cells. Moreover, expression of the *PARD3B* gene in human GBM tissues was increased compared to normal human brain tissues. PARD3B is a polarity and scaffold protein that can regulate the cell cytoskeleton, morphology, and shape and then physiologically control differential cell activities. In addition, PARD3B may contribute to the oncogenesis of colorectal cancer and CNS embryonal tumors [17,18]. In this study, compared to normal human brain tissues, expression of PARD3B mRNA in human GBM tissues was meaningfully elevated. Our data also showed the existence of five AR-specific DNA-binding elements in the 5′ promoter of the *PARD3B* gene. After exposure to testosterone, levels of PARD3B in human glioblastoma cells were induced. Reducing AR activity simultaneously inhibited testosterone-induced PARD3B mRNA expression. Therefore, the testosterone AR–PARD3B axis can stimulate proliferation and colony formation of human glioblastoma cells, resulting in tumorigenesis and successive malignance of human GBM. Targeting such a signaling axis could be potentially applied for treating GBM.

*AR* and *PARD3B* gene expressions are upregulated in human GBM tissues. The data-mining results indicated that expression of the *AR* gene in human GBM tissues was upregulated compared to normal human brains. A previous study showed that levels of testosterone in GBM tissues were also increased [11]. Testosterone is an effective ligand for specific binding to the AR and consequently triggers AR-transducing signals [30]. Our unpublished data demonstrated the effects of testosterone on inducing AR mRNA and protein expressions in human and murine glioblastoma cells. Thus, upregulation of the *AR* gene expression in the microenvironment of human GBM may be due to augmentation of testosterone. In GBM, upregulation of the *AR* gene can alter DNA repair responses, immunity, and the hormone status in the tumor microenvironment [10,11]. In contrast, levels of AR mRNA in pan-TCGA cancer types, including BLCA, CESC, CHOL, HNSC, KICH, LIHC, LUAD, LUSC, READ, THCA, THYM, and UCEC, were downregulated compared to their respective normal tissues. In addition to the involvement of the AR signaling events in tumorigenesis of GBM, the AR-signaling axis was also shown to participate in the oncogenesis of various types of cancers, especially prostate cancer. Consequently, exposure of human U87 MG and GBM8401 glioblastoma cells to testosterone increased cell proliferation. Rapid proliferation is a featured reason explaining GBM malignance and tumor recurrence [1,3]. In parallel, the capabilities of these two types of human glioblastoma cells to produce colonies were concentration- and time-dependently augmented following exposure to testosterone. Results of a clonogenic assay indicated the capability of a single cell to grow into a large colony through clonal expansion [31]. Enhancement of colony formation can occur because of testosterone-induced proliferation of human glioblastoma cells. Fascinatingly, suppressing AR activity with enzalutamide, a specific inhibitor of the AR [25], concurrently attenuated testosterone-induced enhancements of cell proliferation and colony formation in human U87 MG and GBM5401 glioblastoma cells. Accordingly, this study demonstrated the de novo roles of the testosterone AR signaling axis in tumorigenesis of human glioblastomas through stimulating cell proliferation and colony formation.

Similar to *AR* gene expression, expression of PARD3B mRNA in human GBM tissues was significantly increased compared to normal human brains. In addition, in a heatmap of the top 25 gene expressions that were positively correlated with *AR* gene expression in human GBM tissues, *PARD3B* ranked second. Results of a Pearson correlation analysis further showed the coefficient between *AR* and *PARD3B* gene expressions in human GBM tissues was as high as 0.74. Physiologically, PARD3B plays dual roles in working as a polarity protein for producing cell polarity and also as a scaffold protein for shaping the cell cytoskeleton and morphology [14,15]. Dysregulation of cell polarity can lead to migration, invasion, and metastasis of cancer cells [13]. Furthermore, being a scaffold protein, PARD3B connects to downstream intracellular signaling molecules, such as the Hippo-KIBRA/Yap, PI3K/AKT, and TIAM-mediated Rac1 pathways [15]. A change in expression of the *PARD3B* gene may cause deregulation of these transducing signals and subsequently result in oncogenesis. Previous studies reported the roles of PARD3B in neoplasms of colon cancer and CNS embryonic tumors [17,18]. Those findings indicated the potential action of PARD3B as an oncogenic protein in GBM tumorigenesis. Among different Caucasian, African American, and Asian races, positive correlations of *AR* gene expression with *PARD3B* gene expression existed. Results of a supplementary bioinformatics search revealed that there were five predicted AR-specific DNA-binding elements in the 5′ promoter of the *PARD3B* gene. Moreover, our evidence from in vitro models further proved that exposure of human U87 MG and GBM8401 glioblastoma cells, respectively derived from Caucasian and Asian cases [22,23], to testosterone induced PARD3B mRNA expression in concentration- and time-dependent manners. Reducing AR activation by enzalutamide simultaneously inhibited testosterone-induced PARD3B mRNA expression in human glioblastoma cells. Hence, upregulation of *PARD3B* gene expression in human GBM is due to activation of the testosterone AR signaling pathway. More importantly, lowering AR activity resulted in a significant attenuation of testosterone-induced proliferation and colony formation of human glioblastoma cells. Therefore, the testosterone AR–PARD3B signaling axis may contribute to tumorigenesis of human GBM.

The AR-signaling axis-involved regulation of cell proliferation and colony formation in human glioblastoma cells may contribute to tumorigenesis and subsequent malignance of human GBM. The present study demonstrated a positive role of the testosterone AR signaling alliance in the proliferation of human glioblastoma cells. In the process of neoplasm formation, proliferation of initiated tumor cells plays a critical role in the initiation stage and subsequent promotion and progression stages [32]. Defeating proliferation of glioblastoma cells by precisely targeting a molecule of ubiquitin-specific protease, one of the deubiquitinating enzymes, could be a potential strategy for treating GBM [33]. Likewise, rapid proliferation of residual cells after surgical removal of a primary tumor is a leading cause explaining malignance of glioblastomas, poor prognosis of GBM therapy, and tumor recurrence [3,4,5]. Our data showed that the capability of a single U87 MG or GBM8401 glioblastoma cell to grow and extend to a colony was enhanced after exposure to testosterone. The testosterone-induced proliferation of human glioblastoma cells can partially elucidate the enhanced capability for colony formation. Using dietary agents, including flavonoids, pomegranate, carotenoids, sulforaphane, curcumin, resveratrol, berberine, and ginseng, to reduce the capacity of cancer stem cells and cancer cells to form colonies can simultaneously impair cancer stemness, induce apoptosis of cancer cells, and enhance sensitivity of tumors to chemotherapy and radiotherapy in vitro and in vivo [34]. Thus, testosterone AR–PARD3B signaling events may be involved in tumorigenesis and malignance of human GBM through regulating cell proliferation and colony formation.

Human U87 MG and GBM8401 glioblastoma cells have similar responses to stimuli of testosterone and enzalutamide. Human U87 MG and GBM8401 cells are derived from a Caucasian and an Asian GBM patient, respectively [22,23]. Treatment with testosterone induced expression of the *PARD3B* gene in human U87 MG and GBM8401 glioblastoma cells. Subsequently, proliferation and colony formation in these two types of GBM cells were enlarged following exposure to testosterone. In contrast, pretreatment with enzalutamide led to significant attenuations in testosterone-induced PARD3B mRNA expression, cell proliferation, and colony formation in both U87 MG and GBM8401 cells. Even though U87 MG cells had better responses to stimuli of testosterone and enzalutamide than GBM8401, there was no significant differences between these two types of glioblastoma cells. In this study, we have shown upregulation of the *AR* and *PARD3B* gene expressions in GBM patients. Moreover, a positive correlation between *AR* and *PARD3B* gene expression was identified. Our in vitro results showed that suppressing the AR activation concurrently alleviated the testosterone-induced PARD3B mRNA expression, cell proliferation, and colony formation in human glioblastoma cells. Previous studies demonstrated the involvement of the AR signaling axis in tumorigenesis of various types of cancers, including prostate, ovary, bladder, lung, liver, and kidney malignances [8,9]. Fascinatingly, targeting AR signaling is widely investigated for treatment of multiple types of cancers, especially in prostate cancer [9]. Łysiak et al. showed the positive correlation of *AR* gene expression with the DNA repair response in the microenvironment of GBM tissues [10]. Our present results obtained from a bioinformatics search show that there were 5 AR binding elements found in the 5′ promoter region of the *PARD3B* gene. Therefore, the testosterone AR–PARD3B signaling axis may contribute to tumorigenesis and malignance of glioblastomas. Targeting this AR-signaling alliance has the potential for establishing de novo strategies for treatment of GBM.

## 5. Conclusions

Compared to normal human brain tissues, expressions of *AR* and *PARD3B* genes in human GBM tissues were upregulated. In contrast, levels of AR and PARD3B mRNAs in most of TCGA cancer types were downregulated compared to their respective normal tissues. Interestingly, *AR* and *PARD3B* gene expressions in human GBM tissues were positively correlated. Results of a bioinformatics search predicted five AR-specific DNA-binding elements in the 5′ promoter region of the *PARD3B* gene, indicating that AR could transcriptionally regulate expression of the *PARD3B* gene in human GBM tissues. An RNA analysis further showed that exposure of human U87 MG and GBM8401 glioblastoma cells to testosterone induced expression of PARD3B mRNA in concentration- and time-dependent manners. As to the mechanisms, treatment of human U87 MG and GBM8401 glioblastoma cells led to significant augmentations in cell proliferation and colony formation. We further used a loss-of-function strategy by decreasing AR activity using its specific inhibitor, enzalutamide, to identify the positive roles of the AR in regulating *PARD3B* gene expression. In addition, the involvement of the testosterone AR–PARD3B signaling axis in proliferation and colony formation of human glioblastoma cells was successfully confirmed. Rapid proliferation of residual glioblastoma cells and their capability to grow and expand to colonies are tightly linked to tumorigenesis and malignance of GBM. Therefore, this study showed the positive roles of the testosterone AR–PARD3B signaling axis in proliferation and colony formation of human glioblastoma cells and consequently in contributing to tumorigenesis and malignance of glioblastomas. The AR–PARD3B signaling pathway could be targeted for treatment of GBM. In this study, there are certain limitations, including (1) the detailed mechanisms explaining how PARD3B is involved in proliferation and colony formation of human glioblastoma cells should be further investigated; (2) because both U87 MG and GBM8401 cell lines were derived from female GBM patients, the cells from male patients also must be checked; and (3) animal studies should be carried out to confirm our present findings.

## Data Availability

All data generated during this study are included in this article.
